# Protease and *Bacillus coagulans* Supplementation in a Low-Protein Diet Improves Broiler Growth, Promotes Amino Acid Transport Gene Activity, Strengthens Intestinal Barriers, and Alters the Cecal Microbial Composition

**DOI:** 10.3390/ani15020170

**Published:** 2025-01-10

**Authors:** Junlong Niu, Yingying Qiao, Xiaopeng Yang, Xiaoshuang Chen, Hongfei Li, Yongpeng Guo, Wei Zhang, Zhixiang Wang

**Affiliations:** 1 College of Animal Science and Technology, Henan Agricultural University, Zhengzhou 450046, China; niujunlong1234@163.com (J.N.); yxp199804@163.com (X.Y.); 15090252954@163.com (X.C.); lihongfei@stu.henau.edu.cn (H.L.); guoyp@henau.edu.cn (Y.G.); 2 College of Animal Science and Technology, Henan University of Animal Husbandry and Economy, Zhengzhou 450003, China; q18236799337@163.com

**Keywords:** low-protein diet, protease, *Bacillus coagulans*, growth performance, intestinal health

## Abstract

Low-protein (LPRO) diets can effectively reduce feed costs and decrease environmental pollution, making them an important pathway to enhance the sustainability of livestock production. However, they may have adverse effects on the growth performance of broiler chickens, which has limited their widespread application. This study aims to explore the impact of adding protease (PRO) to LPRO diets on the growth performance of broiler chickens, especially under conditions with or without the presence of *Bacillus coagulans* (BC), in order to provide theoretical support for the scientific application and promotion of LPRO feeds. We selected 432 one-day-old broiler chickens and divided them into four treatment groups, which were fed with the control (CON) diet, the LPRO diet, the PRO diet (LPRO diet with added protease), and the PAB diet (PRO diet with added BC). The LPRO group demonstrated decreased growth performance while both PRO and PAB diets resulted in a significant increase (*p* < 0.05). Both PRO and PAB diets significantly enhanced the expression of amino acid transport genes and tight junction genes (*p* < 0.05) and optimized the composition of the intestinal microbiota. Overall, LPRO diets have a detrimental effect on the growth of broiler chickens, while the PRO and PAB diets effectively counteract these negative effects by improving protein digestion, amino acid absorption, and intestinal health.

## Simple Summary

Low-protein (LPRO) diets provide benefits such as reduced feed costs and improved utilization. However, the necessary nutritional data for optimizing these diets are incomplete, and formulation techniques are still developing. Moreover, there is no consensus on the impact of low-protein diets with balanced amino acids on production performance. This study examined the impact of protease and *Bacillus coagulans* on broilers fed LPRO diets, finding that these additives can counteract the negative effects by increasing serum amino acids, upregulating transporter genes, enhancing intestinal barriers, and improving cecal microbiota, offering a basis for promoting LPRO feeds in broiler production.

## 1. Introduction

With the improvement in living standards, the demand for high-quality protein has continuously increased, driving the rapid development of the livestock and poultry industry. However, this growth has also led to a surge in demand for protein feed ingredients, thereby exacerbating the problem of raw material shortages. Therefore, it has become very urgent to seek methods that can effectively reduce the use of protein feed without affecting animal production. Using low-protein (LPRO) diets designed based on the principle of amino acid balance is an effective approach [1,2]. Firstly, LPRO diets can reduce feed expenses, helping enterprises achieve the goal of minimum-cost breeding [3]. Secondly, diets high in crude protein (CP) increase intestinal stress and the risk of disease [4], while LPRO diets can effectively prevent these issues. Furthermore, reducing the protein concentration in feed is the most effective way to reduce nitrogen emissions and alleviate environmental pollution [5]. However, some studies have shown that even LPRO diets supplemented with digestible amino acids cannot maintain production levels [6,7]. Therefore, further theoretical support is needed to advocate and validate the use of LPRO diets in order to overcome these challenges and promote the sustainable development of the livestock industry.

Protease (PRO) and *Bacillus coagulans* (BC) can play important roles in protein digestion. PRO offers several advantages as a feed additive, including a lack of residue and pollution, and is not harmful to either human or animal health. The research by Cowieson and Roos [8] reported that the addition of exogenous PRO to the diet can markedly enhance ileal amino acid digestibility. Similarly, studies by Borda-Molina, et al. [9] have shown that the inclusion of PRO in the diet can notably improve cecal amino acid digestibility. These findings suggest that the use of PRO has a substantial positive impact on protein digestion and amino acid utilization. BC is a rod-shaped facultative anaerobe that exhibits strong stress resistance and maintains good biological activity even in environments with gastric acid and high temperatures during feed processing [10]. The study by Mu and Cong [11] indicates that BC can secrete digestive enzymes and antimicrobial lectins, which promote digestion and absorption in the animal gut. Furthermore, Jäger, et al.’s [12] research further demonstrates that BC can increase beneficial bacteria and reduce harmful bacteria, optimizing the microbial community. These research findings suggest that BC has a significant promotional effect on improving protein digestion and amino acid utilization. Based on these findings, we hypothesize that the addition of BC and PRO to the diet can reduce the requirement for protein while maintaining production efficiency. Consequently, this study uses broiler chickens as a model to investigate the effects of adding PRO and BC to LPRO diets on broiler growth performance, amino acid transport gene expression, intestinal barrier function, and cecal microbial communities. The aim of the study is to provide theoretical support for the scientific application and widespread promotion of LPRO feed.

## 2. Materials and Methods

### 2.1. Ethics Statement

The research was approved by the Animal Care Committee at Henan Agricultural University (Approval No. HNND20190306), with all procedures conducted in compliance with the established guidelines and regulations.

### 2.2. Diets and Diet Analysis

The diets were formulated and produced on site and the chickens were fed employing a two-stage feeding regimen. Starter and grower diets were provided from 0 to 21 and 22 to 42 days of age, respectively. Two experimental feeding programs with differing CP levels were provided as follows: control (CON) diets and LPRO diets containing 2% less dietary CP content in each phase. The CON diets contained 22.60% CP in the starter phase and 20.48% in the grower phase. The diets were formulated in accordance with National Research Council (NRC) [13] standards to fulfill ideal AA requirements, including lysine (Lys), methionine (Met), threonine (Thr), tryptophan (Trp), isoleucine (Ile), valine (Val), and arginine (Arg), while maintaining consistent energy levels throughout each growth phase. Ingredients from a single batch were used to prepare all diets for each developmental stage. The soybean meal proportion in the starter diet was lowered from 33.80 to 27.80% and soybean meal in the grower diet was similarly decreased from 26.30 to 20.40%. Table 1 presents the ingredients and calculated nutrient compositions of the diets.

All diets were analyzed for CP with an Automatic Kjeldahl Nitrogen Determinator (K9860, Hanon, Jinan, China). The diets underwent analysis for overall AA content using ion-exchange chromatography followed by post-column derivatization using ninhydrin. The CP in the diets was hydrolyzed using 6 M HCl at 110 °C prior to column loading. Elution was monitored at 440 nm for Pro and 570 nm for all other AAs [14,15]. Trp levels were measured using HPLC as previously described [16]. The CP and AA contents of the diets are given in Table 2. All proximate analyses were performed at the College of Animal Science and Technology, Henan Agricultural University, Zhengzhou, China. The AA analyses were performed at the College of Agronomy, Henan Agricultural University, Zhengzhou, China.

### 2.3. Animal and Experimental Design

Newly born Arbor Acre broiler chickens (*n* = 432) were randomly assigned to four different treatment groups based on similar body weights. Each group had six replicates, with each replicate consisting of three cages and each cage housing six chickens. The four treatment groups were as follows: (1) the Control (CON) group, fed a conventional diet; (2) the LPRO group, fed an amino acid-balanced LPRO diet; (3) the PRO group, fed an amino acid-balanced LPRO diet supplemented with PRO (at 5 × 10^4^ U/g for a total of 200 g per ton); (4) the Mixture (PAB) group, fed an amino acid-balanced LPRO diet supplemented with PRO (at 5 × 10^4^ U/g for a total of 200 g per ton) and BC (at 1 × 10^11^ CFU/g for a total of 100 g per ton). PRO and BC are both provided by Henan Xinyangshao Biotechnology Co., Ltd. (Zhengzhou, China). Lys, Met, and Thr were specifically added to the LPRO diet to achieve nutritional balance.

The birds were raised in rooms designed with a ventilation system. Each single-tier cage was equipped with a hanging feeder and a nipple drinker line. Birds were placed in cages with an area of 0.84 m^2^ (1.05 m × 0.80 m × 0.50 m) that allowed for an initial stocking density of 0.14 m^2^/bird. From the start until day 7, the lighting intensity was maintained at 35 lux, followed by a reduction to 10 lux from day 8 for the remainder of the study. The photoperiod was set to 23 h at d 1 to d 7 and then to 20 h at d 8 until the end of the experiment. Initially, the chamber temperature was regulated at 33 to 34 °C for the first week, after which it was incrementally lowered to ensure the birds’ comfort. The room temperature was stabilized at 21 °C from the third week and maintained for the study duration. During the trial, broiler chickens had free access to water and feed, while we conducted daily monitoring of the humidity and ammonia levels in the housing.

### 2.4. Growth Performance

On days 21 and 42, the weight of the broilers was measured in triplicate, and feed consumption was recorded weekly. These data were used to calculate the average daily gain (ADG) and the average daily feed intake (ADFI). The feed conversion ratio (FCR) was determined by dividing the ADFI by the ADG. Corrections to these calculations were made based on the weight of any deceased chickens. Broiler mortality rates are detailed in Table S1.

### 2.5. Serum Biochemical Indices and Free AA Concentration

On d 42, blood samples were randomly drawn from the brachial veins of each replicate (*n* = 6/group) using vacutainers and a 19-gauge needle. The blood was centrifuged at 3000× *g* at 4 °C for 10 min to extract serum that was kept at −80 °C prior to use [17]. The levels of total protein (TP), albumin (ALB), urea nitrogen (BUN), and uric acid (UA) were measured using kits from Shandong Boke Biological (Jinan, China). Free AA levels were determined using high-performance liquid chromatography with an LC5090 instrument (Fuli Instruments, Wenling, China) as previously described [18].

### 2.6. Collection of Small Intestine Tissue and Cecal Digesta 

On d 42, the animals were euthanized by cervical dislocation, and sections measuring approximately 2 cm were removed from the mid-ileum, mid-jejunum, and mid-duodenum, rinsed with phosphate-buffered saline (PBS), and preserved in 4% paraformaldehyde. The remaining parts of the duodenum, jejunum, and ileum tissues, as well as the cecal contents, were collected separately and stored at −80 °C.

### 2.7. Intestinal Morphological Characteristics

The intestinal segments in 4% paraformaldehyde were embedded in paraffin, sectioned using a microtome, and stained with hematoxylin-eosin (H&E) and examined using a fully automated BA600-4 biomicroscope (Motic China Group, Xiamen, China). High-resolution images of the slides were captured and 5 each of villi and crypt foci were chosen from each image using the Motic DSAssistant software 2.0 to assess the villus height (VH) and crypt depth (CD). These measurements were used to compute the average values as previously described [19].

### 2.8. Expression of AA Transporters and Tight Junction Proteins

Total RNA from tissues stored at −80 °C was isolated following homogenization using RNAiso Plus (Takara, Dalian, China). RNA concentration and purity were assessed by UV spectroscopy. Reverse transcription was conducted using the Prime Script RT Reagent Kit with gDNA Eraser (Takara). Levels of mRNA were then quantified using real-time PCR following the instructions included with the SYBR Premix Ex Taq kit (Takara) using a CFX96 Optics Module (BioRad, Hercules, CA, USA) and gene-specific primers (Table 3). The β-actin gene was used for internal normalization [20] and relative expression was calculated using the 2^−ΔΔCt^ method and with the instrument’s software [21].

The target gene sequences were retrieved from NCBI’s GenBank database during the primer design process. Specific primers were designed based on these sequences using NCBI’s Primer-BLAST tool. Parameters such as primer length, Tm values, and GC content were adjusted to optimize their performance. After the design was completed, the specificity of the primers was verified through BLAST searches to ensure there was no cross-reactivity with non-target sequences. Preliminary PCR experiments were conducted in the laboratory to validate the efficiency and specificity of the primers, thereby ensuring the accuracy of the experimental results [22].

### 2.9. Microbiome Analysis of Cecal Digesta

Total cecal DNA was isolated using the FastDNA Spin Kit for Soil (MP Biomedicals, Santa Ana, CA, USA) and taxonomic identifications were carried out on a GeneAmp 9700 PCR thermocycler (Applied Biosystems, Waltham, MA, USA) using primers for the hypervariable V3–V4 region of the bacterial 16S rRNA gene. The PCR products were purified and underwent sequencing on an Illumina MiSeq PE300 platform operated in paired-end mode following standard protocols as previously described [23]. The sample data were grouped into operational taxonomic units (OTUs) using QIIME 1.9.1 software, with a similarity threshold of 97% [24]. Subsequently, the OTU table was preprocessed, which involved the removal of OTUs with low abundance and samples, as well as the normalization of the data. Statistical analyses were then conducted, including alpha diversity analysis to assess the diversity within samples and beta diversity analysis to evaluate the correlations and differences between samples [25].

### 2.10. Statistical Analysis

Data analysis was conducted using one-way ANOVA through SPSS version 25.0 followed by Tukey’s Honestly Significant Difference (HSD) test for multiple comparisons to evaluate mean differences among groups. A threshold of *p* < 0.05 was set to denote statistical significance among treatment groups. Results are presented as the mean ± standard error of the mean (SEM).

## 3. Results

### 3.1. Growth Performance and Serum Parameters

The four experimental diets did not differ in their effects on growth performance for the first 21 days of the experiment. However, for the time periods d 22 to 42 and d 1 to 42, birds fed LPRO had reduced ADG (*p* < 0.05) and increased FCR (*p* < 0.05), while PRO and PAB increased ADG (*p* < 0.05) and decreased FCR (*p* < 0.05) compared to birds fed the LPRO and was equal to birds fed the CON diet (Table 4). The serum biochemical analysis revealed that the LPRO diet, when compared to the CON group, significantly reduced the serum levels of UA and BUN in broilers (*p* < 0.05). The diet supplemented with PRO, in comparison to the LPRO group, significantly increased the serum concentrations of TP, GLO, BUN, and UA (*p* < 0.05). Moreover, the diet supplemented with PAB, compared to the LPRO group, significantly elevated the serum UA concentration (*p* < 0.05), aligning it with the levels observed in the control group (Table 5). The serum amino acid analysis demonstrated that the LPRO diet, relative to the CON group, significantly increased the serum concentrations of Val, Leu, and Phe, and significantly decreased the concentration of Ile (*p* < 0.05). In contrast to the LPRO diet, the PRO diet significantly enhanced the serum levels of Thr, Leu, Phe, Glu, Ser, Arg, glycine (Gly), Pro, Ala, and Tyr (*p* < 0.05). The increase in free amino acid concentrations in the serum indicates that PRO promotes the digestion and absorption of dietary protein. However, the PAB diet did not exert a significant influence on these amino acid concentrations (*p* > 0.05, Table 6).

### 3.2. Expression of AA Transporters

**Figure 1:**
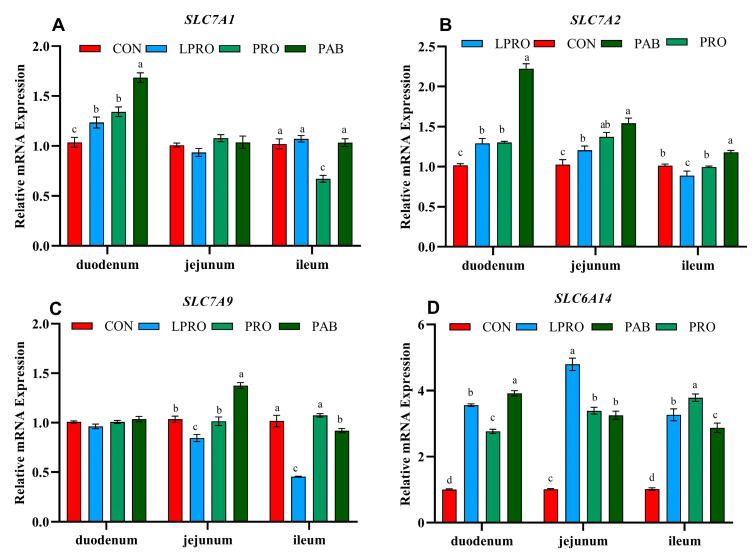
The effect of protease and *Bacillus coagulans* supplementation in a low-protein diet on the relative mRNA expression of amino acid transporters in broilers. (**A**) *SLC7A1*—solute carrier family 7 member 1 (also known as *CAT1*), (**B**) *SLC7A2*—solute carrier family 7 member 2 (also known as *CAT2*), (**C**) *SLC7A9*—solute carrier family 7 member 9 (also known as *b*^0^*^, +AT^*), (**D**) *SLC6A14*—solute carrier family 6 member 14 (also known as *B*^0^*AT*). The error bars represent the SEM values. Statistical significance was set at *p* ≤ 0.05. ^a, b, c^ Means within a row with no common superscripts differ significantly (*p* < 0.05). CON—control diet, LPRO—Low-protein diet, PRO—LPRO + protease diet, PAB—LPRO + protease + *Bacillus coagulans* diet. Dietary protease leave = 5 × 10^4^ U/g, 200 g/t, Dietary *Bacillus coagulans* leave = 1 × 10^11^ CFU/g, 100 g/t.

One indicator of protein metabolism in comparative studies such as ours is alterations in the mRNA expression of AA transporter genes in the intestine. In particular, none of our diets resulted in altered jejunal and ileal expression of *SLC7A1*. However, the LPRO and PAB diets upregulated *SLC7A1* mRNA expression in the duodenum (both *p* < 0.05) compared to birds fed the LPRO diet. When compared with the CON group, the LPRO diet resulted in upregulation of *SLC7A2* mRNA expression in the duodenum and jejunum (*p* < 0.05); however, this gene was downregulated in the ileum (*p* < 0.05) (Figure 1A). PAB upregulated *SLC7A2* mRNA expression in the duodenum, jejunum, and ileum (*p* < 0.05) compared to birds fed LPRO (Figure 1B). In contrast, *SLC7A9* mRNA levels were significantly reduced in the jejunum and ileum with the LPRO diet (*p* < 0.05). Conversely, supplementation with PRO or PAB resulted in a significant increase in *SLC7A9* mRNA expression in the same tissues (*p* < 0.05) when compared to the LPRO group (Figure 1C). Birds fed the LPRO diet showed upregulated *SLC6A14* mRNA expression in the duodenum, jejunum, and ileum (*p* < 0.05), whereas PRO supplementation downregulated *SLC6A14* mRNA expression in the duodenum and jejunum (*p* < 0.05) compared to birds fed the LPRO diet. PAB addition also led to a significant decrease in *SLC6A14* mRNA levels in the jejunum and ileum (*p* < 0.05) (Figure 1D).

### 3.3. Intestinal Morphology

We also found that the diets had no effect on VH, CD, and VH:CD in the jejunum and ileum of the birds. However, the LPRO diet decreased VH and VH:CD in the duodenum (*p* < 0.05). PAB increased the VH and VH:CD and decreased CD in the duodenum (*p* < 0.05) compared to birds fed LPRO. The PRO diet displayed no effect on VH, CD, and VH:CD (*p* > 0.05) compared to birds fed LPRO (Table 7).

### 3.4. Expression of Intestinal Barrier Genes

**Figure 2:**
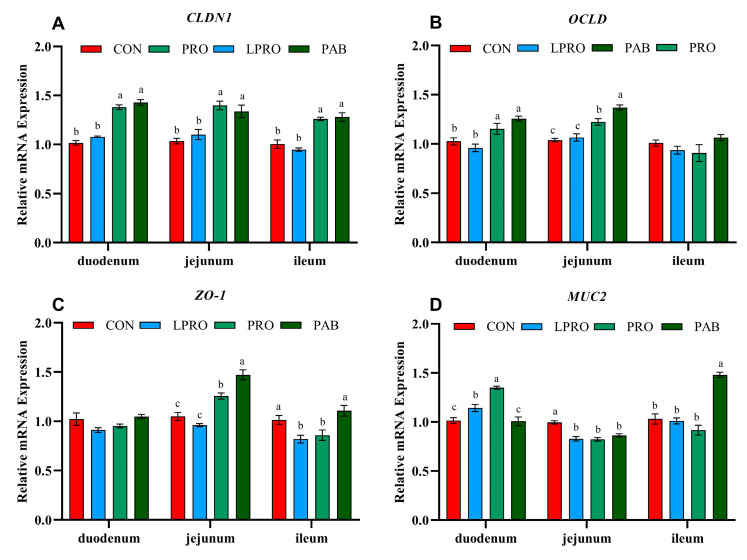
The effect of protease and *Bacillus coagulans* supplementation in a low-protein diet on the relative mRNA expression of the intestinal tract barrier of broilers. (**A**) CLDN1—claudin1; (**B**) OCLD—occludin; (**C**) ZO-1—zonula occludens 1; and (**D**) MUC2—mucin-2. The error bars represent the SEM values. Statistical significance was set at *p* ≤ 0.05. ^a, b, c^ Means within a row with no common superscripts differ significantly (*p* < 0.05). CON—control diet, LPRO—Low-protein diet, PRO—LPRO + protease diet, PAB—LPRO + protease + *Bacillus coagulans* diet. Dietary protease leave = 5 × 10^4^ U/g, 200 g/t, Dietary *Bacillus coagulans* leave = 1 × 10^11^ CFU/g, 100 g/t.

The LPRO diet did not influence the mRNA levels of *CLDN1* and *OCLD* in the duodenum, jejunum, and ileum. However, birds fed LPRO displayed downregulated *ZO-1* mRNA expression in the ileum (*p* < 0.05) and *MUC2* mRNA expression in the jejunum (*p* < 0.05). PRO and PAB upregulated *CLDN1* and *OCLD* mRNA expression in the duodenum and jejunum (*p* < 0.05) compared to birds fed the LPRO diet. PAB upregulated *MUC2* mRNA expression in the ileum (*p* < 0.05) compared to birds fed the LPRO diet (Figure 2).

### 3.5. Cecum Microbiome Analysis

**Figure 3:**
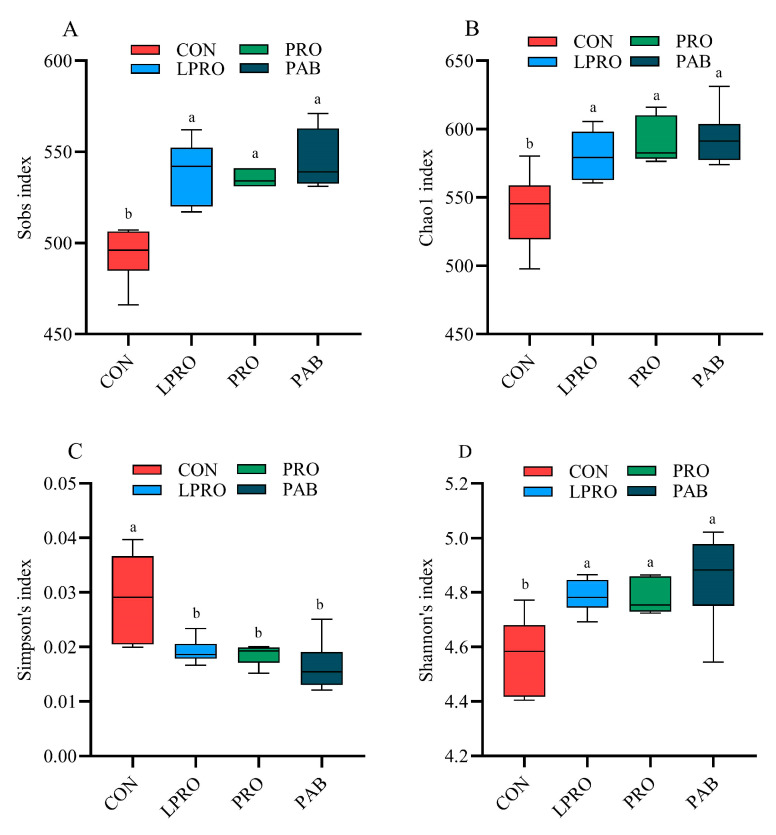
The effect of protease and *Bacillus coagulans* supplementation in a low-protein diet on the alpha diversity of the cecum microbiota in broilers. (**A**) Sobs index; (**B**) Chao index; (**C**) Simpson index; (**D**) Shannon’s index. Statistical significance was set at *p* ≤ 0.05. ^a, b^ Means within a row with no common superscripts differ significantly (*p* < 0.05). CON—control diet, LPRO—Low-protein diet, PRO—LPRO + protease diet, PAB—LPRO + protease + *Bacillus coagulans* diet. Dietary protease leave = 5 × 10^4^ U/g, 200 g/t, Dietary *Bacillus coagulans* leave = 1 × 10^11^ CFU/g, 100 g/t.

**Figure 4:**
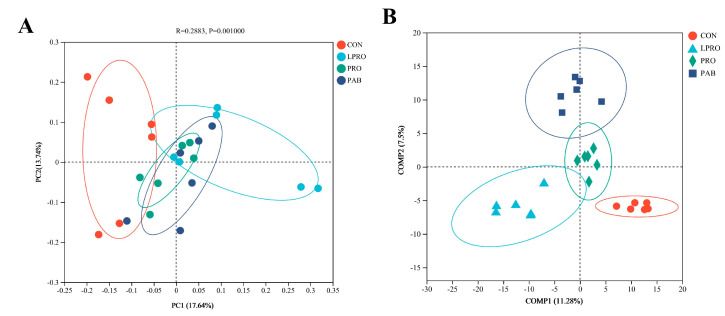
The effect of protease and *Bacillus coagulans* supplementation in a low-protein diet on beta diversity and intergroup similarity of the cecum microbiota in broilers. (**A**) PCoA—principal co-ordinates analysis. The *X*-axis and *Y*-axis represent two selected principal coordinate axes, and the percentages indicate the degree to which the principal coordinate axes explain the compositional differences among samples; the scales on the *X*-axis and *Y*-axis are relative distances and do not have actual meaning; points of different colors or shapes represent samples from different groups, and the closer two sample points are to each other, the more similar the species composition of the two samples is. (**B**) PLS-DA—Partial Least Squares Discriminant Analysis; Different colors or shapes of dots represent sample groups under different environments or conditions, and the scales on the *X*-axis and *Y*-axis are relative distances, which do not have actual significance; Comp1 and Comp2 represent the potential influencing factors for the shifts in microbial composition between the two groups of samples. In the figure, ellipses denote the 95% confidence intervals for sample groups, showing clustering and group separation in principal component space. CON—control diet, LPRO—Low-protein diet, PRO—LPRO + protease diet, PAB—LPRO + protease + *Bacillus coagulans* diet. Dietary protease leave = 5 × 10^4^ U/g, 200 g/t, Dietary *Bacillus coagulans* leave = 1 × 10^11^ CFU/g, 100 g/t.

We also examined the richness and diversity of the microbial communities in the cecal contents of our experimental birds using the α-diversity indices Sobs, Chao, Simpson, and Shannon. Birds fed LPRO displayed increased richness and diversity in their microbial community (*p* < 0.05) based on increased Sobs, Chao, and Shannon indices while the Simpson index decreased. PRO or PAB had no effect on the richness and diversity of the microbial community compared to birds fed LPRO (Figure 3). Community structure changes were then assessed using principal co-ordinates analysis (PCoA) at the OTU level based on the Bray–Curtis algorithm. We found obvious separations between our experimental groups, indicating that the diets changed the structures of the microbial community (Figure 4A). The supervised and analytical partial least squares discriminant analysis (PLS-DA) resulted in the clear segregation of microbial communities among our four groups and revealed substantial variations in community profiles (Figure 4B). These corroborated the results obtained through the PCoA.

**Figure 5:**
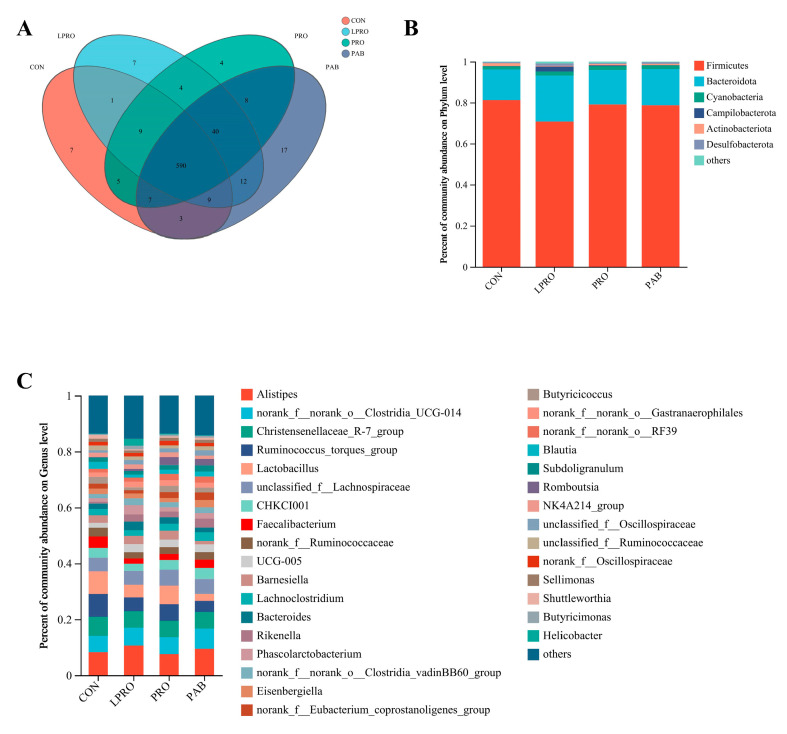
The effect of protease and *Bacillus coagulans* supplementation in a low-protein diet on the species composition of the cecum in broilers. (**A**) Venn Diagram. Percent of community abundance on phylum (**B**) and genus level (**C**). CON—control diet, LPRO—Low-protein diet, PRO—LPRO + protease diet, PAB—LPRO + protease + *Bacillus coagulans* diet. Dietary protease leave = 5 × 10^4^ U/g, 200 g/t, Dietary *Bacillus coagulans* leave = 1 × 10^11^ CFU/g, 100 g/t.

The numbers of OTUs per group were then used to assess the ileal microbiome composition. The CON, LPRO, PRO, and PAB groups possessed 631, 672, 667, and 686 OTUs, respectively, and 590 OTUs were shared among all four groups (Figure 5A). We identified nine bacterial phyla and Firmicutes, Bacteroidota, and Cyanobacteria predominated (Figure 5B). A total of 153 genera were also identified and the dominant bacteria included *Alistipes*, *Clostridia UCG-014*, *Christensenellaceae_R-7*, and *Ruminococcus_torques* (Figure 5C).

**Figure 6:**
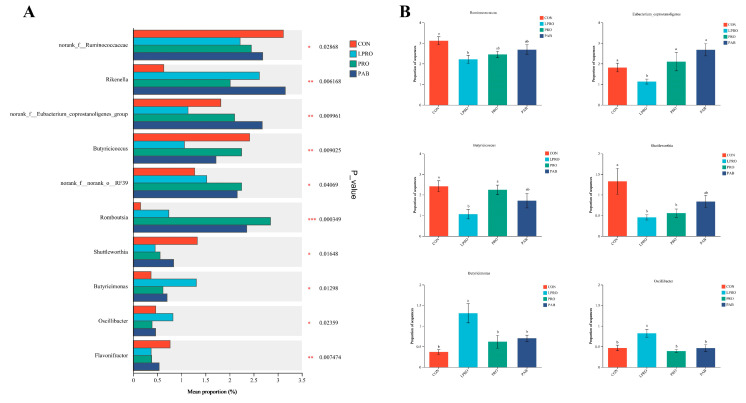
The effect of protease and *Bacillus coagulans* supplementation in a low-protein diet on the relative abundance at the genus level of cecal microbial species in broilers. (**A**) Statistical test for multispecies variation at the genus level; * *p* ≤ 0.05, ** *p* ≤ 0.01, *** *p* ≤ 0.001. (**B**) The average relative abundance of *Ruminococcaceae*, *Eubacterium_coprostanoligenes*, *Butyricicoccus*, *Shuttleworthia*, *Butyricimonas*, and *Oscillibacter*. ^a, b^ Means within a row with no common superscripts differ significantly (*p* < 0.05). CON—Control diet, LPRO—Low-protein diet, PRO—LPRO + protease diet, PAB—LPRO + protease + *Bacillus coagulans* diet. Dietary protease leave = 5 × 10^4^ U/g, 200 g/t, Dietary *Bacillus coagulans* leave = 1 × 10^11^ CFU/g, 100 g/t.

Inter-group differences in the average relative abundance of bacterial genera were analyzed using Kruskal–Wallis H tests to examine the variations in microbial composition between the CON and treatment groups and to identify microbes that exhibit significant differences. At the genus level, we found that the LPRO group exhibited a significant reduction in the levels of *Ruminococcaceae*, *Eubacterium_coprostanoligenes*, *Butyricicoccus*, and *Shuttleworthia* (*p* < 0.05), along with a notable increase in *Butyricimonas* and *Oscillibacter* (both *p* < 0.05). PRO and PAB significantly increased the levels of *Eubacterium_coprostanoligenes* and *Butyricicoccus* (*p* < 0.05), while decreasing those of *Butyricimonas* and *Oscillibacter* (*p* < 0.05) compared to birds fed the LPRO diet (Figure 6A,B).

## 4. Discussion

The potential value of PRO and BC has consistently been a focal point of research. Jabbar, et al. [26]’s study showed that reducing the protein content of feed from 21% to 19%, with the addition of PRO, can promote broiler growth, improve digestibility, and lower feed costs. This finding is further supported by Amer, et al. [27]., who found that increasing PRO supplementation to 200–300 mg/kg significantly enhances broiler growth performance. Elleithy, et al. [28]’s comparative study confirmed that both single-strain and multi-strain Bacillus probiotics effectively increase broilers’ overall body weight and feed conversion ratio. Moreover, Zhang, et al. [29] demonstrated that adding 5 × 10^9^ CFU/kg of BC to the feed, compared to the antibiotic-free base feed, can improve broiler weight and average daily weight gain while also increasing antioxidant enzyme levels, reducing serum pro-inflammatory factor levels, and enhancing anti-inflammatory factor concentrations. Collectively, these studies endorse the scientific rationale behind our chosen doses of PRO and BC, highlighting their potential to improve broiler growth performance. Under the conditions of our experiment, the LPRO diet did not affect growth during the first three weeks, but it significantly reduced development from weeks 4 to 6. Moreover, supplementation with PRO or PAB counteracted the detrimental effects of the LPRO diet on growth performance from weeks 4 to 6. The beneficial effects of PRO were consistent with a previous study that found a 2% reduction in dietary protein levels led to a decrease in growth performance in the later stages of development [30]. We found that the addition of PRO to the LPRO diet returned growth performance to a level comparable to that of the CON group. Danilova and Sharipova [31] have demonstrated that proteolytic enzymes, as feed additives, can enhance nutrient digestibility, boost feed efficiency, and improve both animal growth performance and meat quality. Zhou, et al. [32] have reported that BC facilitates nutrient absorption by producing active enzymes, which in turn improves feed conversion rates. This bacterium also positively impacts energy metabolism through the synthesis of proteins, vitamins, and short-chain fatty acids (SCFAs). Furthermore, BC enhances the intestinal environment and stimulates the secretion of endogenous enzymes, which further improves nutrient digestion rates. Together, these results indicated that when broilers are fed an LPRO diet, it is necessary to add nutritional absorption promoters such as PRO and BC.

Serum protein levels are commonly assessed to determine the animal’s health status and detect conditions such as malnutrition, inflammation, or immune dysfunction [33]. In the current study, serum TP was not affected by the LPRO diet while PRO increased both serum TP and GLO. Similar results have been reported where a 2% reduction in dietary CP concentration had no effect on blood parameters in broilers [34]. In contrast, another study found no impact from LPRO supplementation with PRO on broiler serum TP and this differs from our observations [35]. The inconsistency may be due to the heat stress imposed upon the birds, which differed from our experiments. However, we found that BUN and UA levels were closely linked to dietary composition. A previous study reported a decreasing trend in serum UA concentration in broilers as the dietary CP levels were decreased [36]. We also found that BUN and UA decreased with the LPRO diet compared to CON. Notably, PRO and PAB addition was able to increase both serum BUN and GLO levels, indicating that these additives enhance protein consumption de facto [37].

In the digestive process, AAs interact in intricate ways. Ensuring a balanced AA ratio is essential for sustaining or enhancing animal health and productivity [6]. Serum free AA levels are thus assessment indicators for protein intake and metabolism [38]. In this study, the LPRO diet did not influence serum levels of non-essential AAs in the birds, although it increased serum Val, Leu, and Phe. PRO increased non-essential AAs. PAB did not alter either the levels of essential or non-essential AAs compared to LPRO. Similar experiments have also been reported, where Met-fortified LPRO diets resulted in significant increases in serum Val levels [39]. The increased serum levels of Val, Leu, and Phe were most likely due to the addition of Lys, Met, and Thr to the LPRO diet. The enhancing effect of PRO on protein metabolism was consistent with previous observations [39,40]. In particular, a Leu-fortified LPRO diet significantly reduced body weight gains in broilers [41].

The use of BC as a feed additive can improve the growth performance of livestock and poultry by promoting nutrient absorption [42,43]. However, contrary to our initial expectations, serum AA levels remained unchanged between the PAB and LPRO groups. Nonetheless, the PAB group displayed superior growth performance, which we attribute to enhanced AA metabolism. This assumption is supported by the higher UA levels in the serum of the PAB group since UA is intricately linked to Gly and Ser production [44].

AAs are transported into the body through numerous carriers, which constitutes the basic protein ingestion process [45]. The current study analyzed four specific transporters: the Lys-sensitive transporter SLC7A9, two cationic AA transporters, SLC7A1 and SLC7A2, and the Gly-sensitive transporter SLC6A14. SLC7A9 is expressed in intestinal epithelial cells, serves as an exchange transporter, and is involved in the uptake of Lys and the efflux of Leu [46]. In this study, birds fed the LPRO diet had reduced *SLC7A9* mRNA levels in both the jejunum and ileum while PRO and PAB increased *SLC7A9* mRNA abundance. A previous study reported that dietary supplementation with Lys and Met resulted in increased *SLC7A9* mRNA abundance in the jejunum of piglets [47]. Similar experiments have indicated that Lys-fortified diets significantly increase *SLC7A9* mRNA abundance in the piglet jejunum [48]. It can be inferred that reducing the dietary CP concentration leads to an increase in Leu levels and the addition of PRO or PAB increases the release of Lys, which can reverse the inhibitory effect of Leu on *SLC7A9* [49]. SLC7A1 and SLC7A2 encode high-affinity cationic AA transporters and we found that the LPRO diet increased mRNA levels of *SLC7A1* in the duodenum and *SLC7A2* in the duodenum and jejunum. PAB also increased *SLC7A1* levels in the duodenum and *SLC7A2* levels in the duodenum and jejunum compared to LPRO. Methionine-deficient diets have been shown to upregulate SLC7A1 mRNA expression in the ileum of broilers [50]. *SLC7A2* levels have also been linked to Lys and Arg metabolism since *SLC7A2* promotes Arg intake and shifts the metabolism of cationic AA from NO synthesis to Arg-dependent production of ornithine and urea [51]. This suggests that the PAB-induced increase in *SLC7A1* and *SLC7A2* observed in this study was due to the increased intake of Arg. In contrast, the transporter SLC6A14 acts on a broad range of AA substrates, including Glu and Gly, as well as α- and β-AAs, D- and L-AAs, and AA derivatives [52,53]. *SLC6A14* gene knockout in mice led to significantly reduced serum levels of Gln and Gly and suggested that Glu and Gly were most closely associated with *SLC6A14* gene functions [54]. We found that the LPRO diet upregulated *SLC6A14* mRNA expression in the duodenum, jejunum, and ileum while PRO or PAB downregulated its expression. A similar study indicated that the addition of exogenous PRO significantly decreased the abundance of *SLC6A14* mRNA in the small intestine [55]. In contrast, mouse model studies suggest that SLC6A14 may be involved in the regulation of appetite [53,54]. Gene knockout experiments have also shown that mice lacking the SLC6A14 gene exhibit increased food intake [52]. Therefore, we speculate that the increase in ADFI observed in the PRO and PAB groups of broiler chickens may be associated with the downregulation of SLC6A14.

Intestinal VH and CD are typically used to determine the development status of the intestine [56]. Previous studies have reported that PRO and BC have had a significant impact on improving intestinal development [55,57], while reducing dietary protein levels has a negative effect on VH and CD in the intestine [58]. In our study, similar results were observed, where duodenal VH and VH:CD were reduced when fed with the LPRO diet. However, PAB increased VH and VH:CD in the duodenum and decreased the CD in the duodenum compared to birds fed LPRO. These results suggest that PAB can mitigate the intestinal morphological damage induced by the LPRO diet.

Tight junction proteins and mucin are vital components of the intestinal physical barrier and play a crucial role in maintaining intestinal and overall body health [59,60]. Claudins are essential members of tight junction proteins, which typically interact with other tight junctions such as ZO1 to jointly maintain the integrity of the intestinal barrier [61,62]. In this study, the LPRO diet downregulated the mRNA expression of *ZO-1*, while PRO and PAB upregulated *CLDN1* and *OCLD* expression. A previous study indicated that the use of an AA-fortified LPRO diet still resulted in a decrease in the expression of *CLDN3* and *ZO-2* in the ileum of broilers compared to a normal diet [63]. This was consistent with the results of our study. In addition, mucin secreted by intestinal goblet cells is a key component of the intestinal barrier. In particular, *MUC2* gene expression is essential for the construction of the intestinal mucosa and decreases in its expression are linked to intestinal damage [64]. We found that the LPRO diet downregulated the mRNA expression of *MUC2*, while PAB upregulated it. Broilers receiving a PRO-supplemented diet had significantly upregulated *MUC2* mRNA levels in the jejunum [65]. Dietary BC has also been shown to enhance the mucosal barrier of broilers by increasing the number of intestinal goblet cells [57]. In conclusion, a diet with 2% less CP did have a negative impact on the intestinal barrier of broilers, while PRO and PAB reversed these effects.

Dietary composition is a primary factor that affects the gut microbiota [66]. Specifically, studies have demonstrated a close relationship between dietary protein levels and gut microbial composition [67,68]. In the present study, a significant increase in cecal microbial richness and diversity was noted in the birds fed LPRO, PRO, and PAB diets. Furthermore, at the OTU level, we found a clear separation in the cecal flora between LPRO and CON groups. Similar findings have been reported, where dietary protein levels influenced the composition of the gut microbiota [69]. However, despite differences in dietary composition, the predominant bacterial components were similar in all four treatment groups and *Firmicutes* and *Bacteroidota* were the dominant phyla. This finding was consistent with previous reports [70,71]. The LPRO diet reduced the abundance of Firmicutes and increased Bacteroidota, while PRO and PAB supplementation reversed these effects. Similar results in a study on pigs further support the involvement of *Firmicutes* and *Bacteroidota* in carbohydrate and protein digestion [72]. In conclusion, the addition of PRO or PAB served to restore the microbial changes in the ceca of broilers caused by LPRO.

*Lactobacillus* is a probiotic crucial for intestinal health, aiding in maintaining the integrity of the gut barrier and immune modulation [73]. The *Ruminococcus_torques_group*, another class of microbes associated with gut health and energy metabolism, ferment dietary fiber to produce SCFAs, which are vital for the host’s energy metabolism and intestinal health [74]. In our study, the LPRO diet may have altered the gut environment, reducing the numbers of *Lactobacillus*, which is potentially related to nitrogen utilization and metabolism [75]. However, the LPRO diet promoted the growth of the *Ruminococcus_torques_group*, which is possibly associated with the gut microbiota’s adjustment to the metabolism of protein and fiber [76]. The supplementation of protease and BC can reverse these changes, as BC, as a probiotic, can produce lactic acid in the gut, aiding in the digestion of carbohydrates and proteins [77]. Furthermore, PRO and BC can improve gut health by promoting the growth of beneficial bacteria and the production of SCFAs [12]. In summary, our research results are consistent with the existing scientific literature, indicating that an LPRO diet can significantly affect the composition of the gut microbiota in broiler chickens. By supplementing with PRO and BC, these changes can be regulated, potentially having a positive impact on the growth performance and health of broiler chickens.

## 5. Conclusions

Our research indicates that LPRO diets have adverse effects on the growth of broiler chickens. However, the addition of PRO or PAB can significantly mitigate these negative effects, primarily by promoting protein digestion and amino acid absorption, enhancing intestinal barrier function, and improving the gut microbiota. Given that PRO shows superior performance in promoting amino acid absorption, we recommend prioritizing the use of PRO in practical production.

## Figures and Tables

**Table 1:** Composition of the Diet and Nutrition Levels.

Items ^1^ (%, Unless Otherwise Indicated)	Starter Diets (Days 0 to 21)		Grower Diets (Days 22 to 42)	
	CON	LPRO	CON	LPRO
Corn	54.54	60.85	59.52	65.73
Soybean meal 46%	33.80	27.80	26.30	20.40
Corn gluten meal	5.00	5.00	5.00	5.00
Dicalcium phosphate	1.00	1.00	0.80	0.80
Limestone	1.50	1.50	1.30	1.40
Sodium chloride	0.30	0.30	0.30	0.30
Premix ^2^	0.60	0.60	0.60	0.60
Soybean oil	2.80	2.10	5.60	4.80
L-Lysine monohydrochloride 70%	0.16	0.44	0.34	0.61
DL-Methionine 99%	0.15	0.18	0.09	0.12
Threonine 98%		0.08		0.09
Sodium bicarbonate	0.15	0.15	0.15	0.15
	100.00	100.00	100.00	100.00
Calculated nutrient levels				
ME (MJ/kg)	12.52	12.54	13.43	13.40
Ca	0.91	0.89	0.78	0.80
Available phosphorus	0.70	0.68	0.62	0.60

^1^ CON—control diet, LPRO—Low-protein diet. ^2^ The premix supplied the following per kilogram of diet: from 1 to 21 d, vitamin A, 12,000 IU; vitamin D3, 3500 IU; vitamin E, 60 IU; vitamin K3, 4 mg; vitamin B1, 2.5 mg; vitamin B2, 10 mg; VB6, 6 mg; VB12, 8 μg; biotin, 0.8 mg; folic acid, 10 mg; D-pantothenic acid, 40 mg; nicotinic acid, 75 mg; choline, 700 mg; Zn, 90 mg; Fe, 110 mg; Cu, 20 mg; Mn, 100 mg; Se, 0.3 mg; I, 0.5 mg; phytase, 0.1 g. From 22 to 42 d, vitamin A, 10,000 IU; vitamin D3, 3000 IU; vitamin E, 50 IU; vitamin K3, 3.5 mg; vitamin B1, 2 mg; vitamin B2, 10 mg; VB6, 5 mg; VB12, 6 μg; biotin, 0.6 mg; folic acid, 8 mg; D-pantothenic acid, 20 mg; nicotinic acid, 60 mg; choline, 600 mg; Zn, 80 mg; Fe, 100 mg; Cu, 15 mg; Mn, 80 mg; Se, 0.3 mg; I, 0.5 mg; phytase, 0.1 g.

**Table 2:** Calculated and analyzed crude protein and amino acids of diets.

Items ^1^	Starter Diets (Days 0 to 21)		Grower Diets (Days 22 to 42)	
	CON	LPRO	CON	LPRO
Calculated nutrient levels				
CP	23.02	20.88	20.04	18.12
Total Lys%	1.21	1.26	1.15	1.19
Total Met + Cys%	0.89	0.87	0.75	0.73
Total Met%	0.52	0.53	0.43	0.43
Total Thr%	0.84	0.84	0.73	0.74
Total Val%	1.05	0.94	0.91	0.81
Total Ile%	0.97	0.87	0.83	0.73
Total Arg%	1.45	1.27	1.21	1.04
Total Trp%	0.24	0.21	0.20	0.17
Total His%	0.59	0.53	0.51	0.46
Total Leu%	2.19	2.05	1.99	1.85
Total Phe%	1.15	1.04	1.00	0.89
Total Tyr%	0.83	0.76	0.73	0.66
SID ^2^ Lys%	1.01	1.06	0.94	0.98
SID Met + Cys%	0.75	0.73	0.62	0.61
SID Met%	0.46	0.46	0.36	0.37
SID Thr%	0.68	0.68	0.58	0.59
SID Val%	0.91	0.82	0.77	0.69
SID Ile%	0.84	0.76	0.71	0.63
SID Arg%	1.22	1.07	0.99	0.85
SID Trp%	0.19	0.17	0.15	0.13
SID His%	0.51	0.47	0.44	0.39
SID Leu%	1.86	1.76	1.65	1.55
SID Phe%	0.98	0.89	0.83	0.75
Analyzed nutrient levels				
CP	23.01	20.83	20.11	18.01
Total Lys%	1.19	1.23	1.13	1.15
Total Met + Cys%	0.87	0.88	0.78	0.74
Total Met%	0.49	0.51	0.41	0.41
Total Thr%	0.85	0.85	0.73	0.72
Total Val%	1.17	1.04	0.93	0.88
Total Ile%	0.95	0.86	0.87	0.77
Total Arg%	1.43	1.29	1.18	1.05
Total Trp%	0.25	0.22	0.20	0.17
Total His%	0.56	0.51	0.51	0.47
Total Leu%	2.10	2.00	1.94	1.80
Total Phe%	1.05	0.94	0.87	0.78
Total Tyr%	0.82	0.75	0.71	0.65

^1^ CON—Control diet, LPRO—Low-protein diet; CP—crude protein, Lys—lysine, Met—methionine, Thr—threonine, Val—valine, Ile—isoleucine, Arg—arginine, Trp—tryptophan, His—histidine, Leu—leucine, Phe—phenylalanine, Tyr—tyrosine. ^2^ SID—Standardized ileal digestibility, EVONIK Industries AminoDat 5.0 (2016).

**Table 3:** Primers used for quantitative real-time PCR.

Gene Symbol ^1^	Accession Number	Primer 5′-3′	
*ACT*	NM_205518.2	F ^2^	CCAGCCATGTATGTAGCCATCCAG
		R ^3^	GGTAACACCATCACCAGAGTCCATC
*SLC7A1*	NM_001398060.1	F	CAAGAGGAAAACTCCAGTAATTGCA
		R	AAGTCGAAGAGGAAGGCCATAA
*SLC7A2*	XM_046916231.1	F	TGCTCGCGTTCCCAAGA
		R	GGCCCACAGTTCACCAACAG
*SLC7A9*	XM_046925532.1	F	TGGCTCAGGCATCTTTGTTTCCC
		R	ACAGGCTGCCCAGATGGTTAAAC
*SLC6A14*	XM_040670974.2	F	TTGATGGAGGCAGAGGTTTGGAAAG
		R	AGCATCCGAGTAGCAGTTGTTGTG
*ZO1*	XM_040680630.1	F	GCCTACTGCTGCTCCTTACAACTC
		R	GCTGGATCTATATGCGGCGGTAAG
*CLDN1*	NM_001013611.2	F	ACACCCGTTAACACCAGATTT
		R	GCATTTTTGGGGTAGCCTCG
*OCLDN*	NM_205128.1	F	GATGGACAGCATCAACGACC
		R	CTTGCTTTGGTAGTCTGGGC
*MUC2*	XM_040673077.1	F	ATTGAAGCCAGCAATGGTGT
		R	TGACATCAGGGCACACAGAT

^1^ *ACT*—Beta-actin; *SLC7A1*—solute carrier family 7 member 1 (also known as *CAT1*), *SLC7A2*—solute carrier family 7 member 2 (also known as *CAT2*), *SLC7A9*—solute carrier family 7 member 9 (also known as *b*^0*,+*^*AT*), *SLC6A14*—solute carrier family 6 member 14 (also known as *B*^0^*AT*), *ZO1*—zonula occludens 1, *CLDN1*—claudin1, *OCLDN*—occludine, *MUC2*—mucin-2. ^2^ F—Forward. ^3^ R—Reverse.

**Table 4:** The effect of protease and *Bacillus coagulans* supplementation in a low-protein diet on growth performance in broilers.

Items ^1^	CON	LPRO	PRO	PAB	SEM	*p*-Value
1 to 21 d						
ADG	28.57	29.11	28.97	28.84	1.032	0.851
ADFI	37.97	37.58	37.38	36.78	1.351	0.511
FCR	1.33	1.29	1.29	1.28	0.035	0.051
22 to 42						
ADG	69.72 ^a^	62.84 ^b^	70.90 ^a^	71.07 ^a^	4.089	<0.001
ADFI	108.09 ^b^	108.28 ^b^	113.55 ^a^	112.11 ^ab^	3.976	0.023
FCR	1.55 ^b^	1.72 ^a^	1.60 ^b^	1.58 ^b^	0.077	<0.001
1 to 42 d						
ADG	49.15 ^a^	45.97 ^b^	49.93 ^a^	49.95 ^a^	2.209	0.001
ADFI	73.03	73.29	75.85	74.61	2.339	0.129
FCR	1.49 ^b^	1.59 ^a^	1.52 ^b^	1.49 ^b^	0.052	<0.001

^a, b^ Means within a row with no common superscripts differ significantly (*p* < 0.05). ^1^ CON—control diet, LPRO—Low-protein diet, PRO—LPRO + protease diet, PAB—LPRO + protease + *Bacillus coagulans* diet, SEM—the standard error of the mean; ADG—average daily gain (g/d), ADFI—average daily feed intake (g/d), FCR—feed conversion ratio (g/g); Dietary protease leave = 5 × 10^4^ U/g, 200 g/t; Dietary *Bacillus coagulans* leave = 1 × 10^11^ CFU/g, 100 g/t; Data were presented as the mean and SEM of six broilers in each group (*n* = 6).

**Table 5:** The effect of protease and *Bacillus coagulans* supplementation in a low-protein diet on serum biochemical parameters in broilers.

Item ^1^	CON	LPRO	PRO	PAB	SEM	*p*-Value
TP	36.28 ^b^	36.76 ^b^	42.82 ^a^	34.43 ^b^	0.870	0.001
ALB	14.54 ^b^	15.37 ^ab^	16.03 ^a^	14.30 ^b^	0.252	0.047
GLO	22.25 ^bc^	23.02 ^b^	26.52 ^a^	20.45 ^c^	0.596	<0.001
BUN	0.66 ^a^	0.53 ^b^	0.62 ^a^	0.54 ^b^	0.015	0.001
UA	232.84 ^a^	138.16 ^c^	156.77 ^b^	248.91 ^a^	10.331	<0.001

^a, b, c^ Means within a row with no common superscripts differ significantly (*p* < 0.05). ^1^ TP—total protein (g/L), ALB—albumin (g/L), GLO—globulin (g/L), BUN—urea nitrogen (mmol/L), UA—uric acid (umol/L); CON—control diet, LPRO—Low-protein diet, PRO—LPRO + protease diet, PAB—LPRO + protease + *Bacillus coagulans* diet, SEM—the standard error of the mean; Dietary protease leave = 5 × 10^4^ U/g, 200 g/t; Dietary *Bacillus coagulans* leave = 1 × 10^11^ CFU/g, 100 g/t; Data were presented as the mean and SEM of six broilers in each group (*n* = 6). GLO = TP minus ALB.

**Table 6:** The effect of protease and *Bacillus coagulans* supplementation in a low-protein diet on the concentration of free amino acids in serum in broilers.

Items ^1^	CON	LPRO	PRO	PAB	SEM	*p*-Value
Indispensable						
Thr	1.78 ^b^	1.96 ^b^	2.26 ^a^	1.89 ^b^	0.048	<0.001
Val	2.08 ^b^	2.35 ^a^	2.38 ^a^	2.16 ^b^	0.039	0.005
Ile	1.77 ^a^	1.31 ^c^	1.54 ^b^	1.36 ^c^	0.044	<0.001
Leu	3.05 ^c^	4.72 ^b^	5.50 ^a^	4.75 ^b^	0.201	<0.001
Phe	1.28 ^c^	1.78 ^b^	1.97 ^a^	1.77 ^b^	0.057	<0.001
Dispensable						
Asp	3.21	3.22	3.56	3.30	0.061	0.137
Glu	5.01 ^b^	5.17 ^b^	6.00 ^a^	5.25 ^b^	0.110	0.002
Ser	2.26 ^b^	2.44 ^b^	2.66 ^a^	2.23 ^b^	0.049	0.001
Arg	2.30 ^b^	2.38 ^b^	2.67 ^a^	2.24 ^b^	0.053	0.011
Gly	1.30 ^b^	1.34 ^b^	1.51^a^	1.25 ^b^	0.029	0.002
Pro	1.80 ^b^	1.90 ^b^	2.32 ^a^	1.95 ^b^	0.051	<0.001
Ala	1.83 ^b^	1.85 ^b^	2.19 ^a^	1.89 ^b^	0.044	0.004
Cys	0.90 ^a^	0.62 ^b^	0.64 ^b^	0.56 ^c^	0.028	<0.001
His	4.22 ^b^	4.30 ^b^	4.73 ^a^	4.03 ^b^	0.078	0.004
Tyr	1.43 ^b^	1.59 ^a^	1.70 ^a^	1.44 ^b^	0.031	0.001

^a, b, c^ Means within a row with no common superscripts differ significantly (*p* < 0.05). ^1^ Thr—threonine (mg/mL), Val—valine (mg/mL), Ile—isoleucine (mg/mL), Leu—leucine (mg/mL), Phe—phenylalanine (mg/mL), Asp—aspartic acid (mg/mL), Glu—glutamic acid (mg/mL), Ser—serine (mg/mL), Arg—arginine (mg/mL), Gly—glycine (mg/mL), Pro—proline (mg/mL), Ala—alanine (mg/mL), Cys—cysteine (mg/mL), His—histidine (mg/mL), Tyr—tyrosine (mg/mL); CON—control diet, LPRO—Low-protein diet, PRO—LPRO + protease diet, PAB—LPRO + protease + *Bacillus coagulans* diet, SEM—the standard error of the mean; Dietary protease leave = 5 × 10^4^ U/g, 200 g/t, Dietary *Bacillus coagulans* leave = 1 × 10^11^ CFU/g, 100 g/t; Data were presented as the mean and SEM of six broilers in each group (*n* = 6).

**Table 7:** The effect of protease and *Bacillus coagulans* supplementation in a low-protein diet on small intestinal morphology in broilers.

Items ^1^	CON	LPRO	PRO	PAB	SEM	*p*-Value
Duodenum						
VH	1675.73 ^a^	1291.00 ^b^	1450.60 ^ab^	1591.17 ^a^	49.291	0.020
CD	181.37 ^a^	186.97 ^a^	169.90 ^a^	127.68 ^b^	5.812	<0.001
VH:CD	9.30 ^b^	6.93 ^c^	8.74 ^bc^	12.53 ^a^	0.530	<0.001
Jejunum						
VH	1224.05	1206.61	1234.30	1268.73	33.219	0.936
CD	151.31	167.63	165.15	144.83	3.892	0.106
VH:CD	8.18	7.27	7.51	8.84	0.276	0.178
Ileum						
VH	968.82	884.44	949.80	935.80	13.998	0.170
CD	127.84	137.35	128.85	122.62	2.404	0.185
VH:CD	7.59	6.47	7.40	7.75	0.189	0.067

^a, b, c^ Means within a row with no common superscripts differ significantly (*p* < 0.05). ^1^ VH—villus height (μm), CD—crypt depth (μm); CON—control diet, LPRO—Low-protein diet, PRO—LPRO + protease diet, PAB—LPRO + protease + *Bacillus coagulans* diet, SEM—the standard error of the mean; Dietary protease leave = 5 × 10^4^ U/g, 200 g/t, Dietary *Bacillus coagulans* leave = 1 × 10^11^ CFU/g, 100 g/t; Data were presented as the mean and SEM of six broilers in each group (*n* = 6).

## Data Availability

All data used in this study are available from the authors upon reasonable request.

## Supplementary Materials

The following supporting information can be downloaded at: https://www.mdpi.com/article/10.3390/ani15020170/s1, Table S1: The effect of protease and *Bacillus coagulans* supplementation to a low-protein diet on mortality rate in broilers.

## References

[1] Hou Y., Wu Z., Dai Z., Wang G., Wu G. (2017). Protein hydrolysates in animal nutrition: Industrial production, bioactive peptides, and functional significance. J. Anim. Sci. Biotechnol..

[2] Kidd M.T., Maynard C.W., Mullenix G.J. (2021). Progress of amino acid nutrition for diet protein reduction in poultry. J. Anim. Sci. Biotechnol..

[3] Fraps G.S. (1943). Relation of the protein, fat, and energy of the ration to the composition of chickens. Poult. Sci..

[4] Kitada M., Ogura Y., Monno I., Koya D. (2019). The impact of dietary protein intake on longevity and metabolic health. EBioMedicine.

[5] van Emous R.A., Winkel A., Aarnink A.J.A. (2019). Effects of dietary crude protein levels on ammonia emission, litter and manure composition, n losses, and water intake in broiler breeders. Poult. Sci..

[6] Parenteau I.A., Stevenson M., Kiarie E.G. (2020). Egg production and quality responses to increasing isoleucine supplementation in shaver white hens fed a low crude protein corn-soybean meal diet fortified with synthetic amino acids between 20 and 46 weeks of age. Poult. Sci..

[7] Ajao A.M., White D., Kim W.K., Olukosi O.A. (2022). Partial replacement of soybean meal with canola meal or corn ddgs in low-protein diets supplemented with crystalline amino acids-effect on growth performance, whole-body composition, and litter characteristics. Animals.

[8] Cowieson A.J., Roos F.F. (2016). Toward optimal value creation through the application of exogenous mono-component protease in the diets of non-ruminants. Anim. Feed. Sci. Technol..

[9] Borda-Molina D., Zuber T., Siegert W., Camarinha-Silva A., Feuerstein D., Rodehutscord M. (2019). Effects of protease and phytase supplements on small intestinal microbiota and amino acid digestibility in broiler chickens. Poult. Sci..

[10] Konuray G., Erginkaya Z. (2018). Potential use of bacillus coagulans in the food industry. Foods.

[11] Mu Y., Cong Y. (2019). Bacillus coagulans and its applications in medicine. Benef. Microbes.

[12] Jäger R., Purpura M., Farmer S., Cash H.A., Keller D. (2018). Probiotic bacillus coagulans gbi-30, 6086 improves protein absorption and utilization. Probiotics Antimicrob. Proteins.

[13] National Research Council (NRC) (1994). Nutrient Requirements of Poultry.

[14] Directive C. (1998). Establishing community methods for 434 the determination of amino acids, crude oils and fats, and olan-quindox in feeding stuff and amending directive. Off. J. Eur. Communities L.

[15] Llames C.R., Fontaine J. (1994). Determination of amino acids in feeds collaborative study. J. AOAC Int..

[16] Directive C. (2000). Establishing community methods for the determination of vitamin a, vitamin e and tryptophan, annex part c. Offic. J..

[17] Cheng Q., Xia Y., Yi D., Hou Y., Duan R., Guo S., Ding B. (2019). The intestinal cinnamaldehyde release and antioxidative capacity of broiler chickens fed diets supplemented with coated oleum cinnamomi. J. Appl. Poult. Res..

[18] Morales A., Buenabad L., Castillo G., Vázquez L., Espinoza S., Htoo J.K., Cervantes M. (2016). Dietary levels of protein and free amino acids affect pancreatic proteases activities, amino acids transporters expression and serum amino acid concentrations in starter pigs. J. Anim. Physiol. Anim. Nutr..

[19] Zheng Y.W., Zhang J.Y., Zhou H.B., Guo Y.P., Ma Q.G., Ji C., Zhao L.H. (2020). Effects of dietary pyrroloquinoline quinone disodium supplementation on inflammatory responses, oxidative stress, and intestinal morphology in broiler chickens challenged with lipopolysaccharide. Poult. Sci..

[20] Pfaffl M.W., Tichopad A., Prgomet C., Neuvians T.P. (2004). Determination of stable housekeeping genes, differentially regulated target genes and sample integrity: Bestkeeper--excel-based tool using pair-wise correlations. Biotechnol. Lett..

[21] Zhang X., Xu H., Gong L., Wang J., Fu J., Lv Z., Zhou L., Li X., Liu Q., Xia P. (2024). Mannanase improves the growth performance of broilers by alleviating inflammation of the intestinal epithelium and improving intestinal microbiota. Anim. Nutr..

[22] Dwivedi-Yu J.A., Oppler Z.J., Mitchell M.W., Song Y.S., Brisson D. (2023). A fast machine-learning-guided primer design pipeline for selective whole genome amplification. PLoS Comput. Biol..

[23] Yang Z., Xu M., Li Q., Wang T., Zhang B., Zhao H., Fu J. (2021). The beneficial effects of polysaccharide obtained from persimmon (diospyros kaki l.) on the proliferation of lactobacillus and gut microbiota. Int. J. Biol. Macromol..

[24] Edgar R.C. (2013). Uparse: Highly accurate otu sequences from microbial amplicon reads. Nat. Methods.

[25] Wang Q., Garrity G.M., Tiedje J.M., Cole J.R. (2007). Naive bayesian classifier for rapid assignment of rrna sequences into the new bacterial taxonomy. Appl. Environ. Microbiol..

[26] Jabbar A., Tahir M., Alhidary I.A., Abdelrahman M.A., Albadani H., Khan R.U., Selvaggi M., Laudadio V., Tufarelli V. (2021). Impact of microbial protease enzyme and dietary crude protein levels on growth and nutrients digestibility in broilers over 15–28 days. Animals.

[27] Amer S.A., Beheiry R.R., Abdel Fattah D.M., Roushdy E.M., Hassan F.A.M., Ismail T.A., Zaitoun N.M.A., Abo-Elmaaty A.M.A., Metwally A.E. (2021). Effects of different feeding regimens with protease supplementation on growth, amino acid digestibility, economic efficiency, blood biochemical parameters, and intestinal histology in broiler chickens. BMC Vet. Res..

[28] Elleithy E.M.M., Bawish B.M., Kamel S., Ismael E., Bashir D.W., Hamza D., Fahmy K.N.E. (2023). Influence of dietary bacillus coagulans and/or bacillus licheniformis-based probiotics on performance, gut health, gene expression, and litter quality of broiler chickens. Trop. Anim. Health Prod..

[29] Zhang B., Zhang H., Yu Y., Zhang R., Wu Y., Yue M., Yang C. (2021). Effects of bacillus coagulans on growth performance, antioxidant capacity, immunity function, and gut health in broilers. Poult. Sci..

[30] Wang T., Ling H., Zhang W., Zhou Y., Li Y., Hu Y., Peng N., Zhao S. (2022). Protease or clostridium butyricum addition to a low-protein diet improves broiler growth performance. Appl. Microbiol. Biotechnol..

[31] Danilova I., Sharipova M. (2020). The practical potential of bacilli and their enzymes for industrial production. Front. Microbiol..

[32] Zhou Y., Zeng Z., Xu Y., Ying J., Wang B., Majeed M., Majeed S., Pande A., Li W. (2020). Application of bacillus coagulans in animal husbandry and its underlying mechanisms. Animals.

[33] Batavani R.A., Ansari M.H., Asri S. (2006). Concentrations of serum total protein and protein fractions during diestrus and pregnancy in makuii ewes. Comp. Clin. Pathol..

[34] Saleh A.A., Amber K.A., Soliman M.M., Soliman M.Y., Morsy W.A., Shukry M., Alzawqari M.H. (2021). Effect of low protein diets with amino acids supplementation on growth performance, carcass traits, blood parameters and muscle amino acids profile in broiler chickens under high ambient temperature. Agriculture.

[35] Lin Law F., Idrus Z., Soleimani Farjam A., Juan Boo L., Awad E.A. (2019). Effects of protease supplementation of low protein and/or energy diets on growth performance and blood parameters in broiler chickens under heat stress condition. Ital. J. Anim. Sci..

[36] Namroud N.F., Shivazad M., Zaghari M. (2008). Effects of fortifying low crude protein diet with crystalline amino acids on performance, blood ammonia level, and excreta characteristics of broiler chicks. Poult. Sci..

[37] Rubio L.A., Brenes A., Centeno C. (2003). Effects of feeding growing broiler chickens with practical diets containing sweet lupin (lupinus angustifolius) seed meal. Br. Poult. Sci..

[38] Truong H.H., Chrystal P.V., Moss A.F., Selle P.H., Liu S.Y. (2017). Rapid protein disappearance rates along the small intestine advantage poultry performance and influence the post-enteral availability of amino acids. Br. J. Nutr..

[39] Chrystal P.V., Moss A.F., Khoddami A., Naranjo V.D., Selle P.H., Liu S.Y. (2020). Impacts of reduced-crude protein diets on key parameters in male broiler chickens offered maize-based diets. Poult. Sci..

[40] Safari H., Mohit A., Mohiti-Asli M. (2024). Feather meal processing methods impact the production parameters, blood biochemical indices, gut function, and hepatic enzyme activity in broilers. J. Anim. Sci..

[41] D’Mello J.P., Lewis D. (1970). Amino acid interactions in chick nutrition. 2. Interrelationships between leucine, isoleucine and valine. Br. Poult. Sci..

[42] Li C.L., Wang J., Zhang H.J., Wu S.G., Hui Q.R., Yang C.B., Fang R.J., Qi G.H. (2018). Intestinal morphologic and microbiota responses to dietary bacillus spp. In a broiler chicken model. Front. Physiol..

[43] Sen S., Ingale S.L., Kim Y.W., Kim J.S., Kim K.H., Lohakare J.D., Kim E.K., Kim H.S., Ryu M.H., Kwon I.K. (2012). Effect of supplementation of bacillus subtilis ls 1-2 to broiler diets on growth performance, nutrient retention, caecal microbiology and small intestinal morphology. Res. Vet. Sci..

[44] Kriseldi R., Tillman P.B., Jiang Z., Dozier W.A. (2018). Effects of feeding reduced crude protein diets on growth performance, nitrogen excretion, and plasma uric acid concentration of broiler chicks during the starter period. Poult. Sci..

[45] Fotiadis D., Kanai Y., Palacín M. (2013). The slc3 and slc7 families of amino acid transporters. Mol. Asp. Med..

[46] Pineda M., Wagner C.A., Bröer A., Stehberger P.A., Kaltenbach S., Gelpí J.L., Martín Del Río R., Zorzano A., Palacín M., Lang F. (2004). Cystinuria-specific rbat(r365w) mutation reveals two translocation pathways in the amino acid transporter rbat-b0,+at. Biochem. J..

[47] García-Villalobos H., Morales-Trejo A., Araiza-Piña B.A., Htoo J.K., Cervantes-Ramírez M. (2012). Effects of dietary protein and amino acid levels on the expression of selected cationic amino acid transporters and serum amino acid concentration in growing pigs. Arch. Anim. Nutr..

[48] He L., Yang H., Hou Y., Li T., Fang J., Zhou X., Yin Y., Wu L., Nyachoti M., Wu G. (2013). Effects of dietary l-lysine intake on the intestinal mucosa and expression of cat genes in weaned piglets. Amino Acids.

[49] Morales A., Barrera M.A., Araiza A.B., Zijlstra R.T., Bernal H., Cervantes M. (2013). Effect of excess levels of lysine and leucine in wheat-based, amino acid-fortified diets on the mrna expression of two selected cationic amino acid transporters in pigs. J. Anim. Physiol. Anim. Nutr..

[50] Fagundes N.S., Milfort M.C., Williams S.M., Da Costa M.J., Fuller A.L., Menten J.F., Rekaya R., Aggrey S.E. (2020). Dietary methionine level alters growth, digestibility, and gene expression of amino acid transporters in meat-type chickens. Poult. Sci..

[51] Visigalli R., Barilli A., Parolari A., Sala R., Rotoli B.M., Bussolati O., Gazzola G.C., Dall’Asta V. (2010). Regulation of arginine transport and metabolism by protein kinase calpha in endothelial cells: Stimulation of cat2 transporters and arginase activity. J. Mol. Cell Cardiol..

[52] Hatanaka T., Huang W., Nakanishi T., Bridges C.C., Smith S.B., Prasad P.D., Ganapathy M.E., Ganapathy V. (2002). Transport of d-serine via the amino acid transporter atb(0,+) expressed in the colon. Biochem. Biophys. Res. Commun..

[53] Hatanaka T., Haramura M., Fei Y.J., Miyauchi S., Bridges C.C., Ganapathy P.S., Smith S.B., Ganapathy V., Ganapathy M.E. (2004). Transport of amino acid-based prodrugs by the na+- and cl(-) -coupled amino acid transporter atb0,+ and expression of the transporter in tissues amenable for drug delivery. J. Pharmacol. Exp. Ther..

[54] Sivaprakasam S., Sikder M.O.F., Ramalingam L., Kaur G., Dufour J.M., Moustaid-Moussa N., Wachtel M.S., Ganapathy V. (2021). Slc6a14 deficiency is linked to obesity, fatty liver, and metabolic syndrome but only under conditions of a high-fat diet. Biochim. Biophys. Acta Mol. Basis Dis..

[55] Lu P., Choi J., Yang C., Mogire M., Liu S., Lahaye L., Adewole D., Rodas-Gonzalez A., Yang C. (2020). Effects of antibiotic growth promoter and dietary protease on growth performance, apparent ileal digestibility, intestinal morphology, meat quality, and intestinal gene expression in broiler chickens: A comparison. J. Anim. Sci..

[56] Incharoen T., Yamauchi K.-e., Erikawa T., Gotoh H. (2010). Histology of intestinal villi and epithelial cells in chickens fed low-crude protein or low-crude fat diets. Ital. J. Anim. Sci..

[57] Liu C., Radebe S.M., Zhang H., Jia J., Xie S., Shi M., Yu Q. (2022). Effect of bacillus coagulans on maintaining the integrity intestinal mucosal barrier in broilers. Vet. Microbiol..

[58] Allameh S., Toghyani M. (2019). Effect of dietary valine supplementation to low protein diets on performance, intestinal morphology and immune responses in broiler chickens. Livest. Sci..

[59] Otani T., Furuse M. (2020). Tight junction structure and function revisited. Trends Cell Biol..

[60] Cox K.E., Liu S., Lwin T.M., Hoffman R.M., Batra S.K., Bouvet M. (2023). The mucin family of proteins: Candidates as potential biomarkers for colon cancer. Cancers.

[61] Guo W., Wang P., Liu Z.H., Ye P. (2018). Analysis of differential expression of tight junction proteins in cultured oral epithelial cells altered by porphyromonas gingivalis, porphyromonas gingivalis lipopolysaccharide, and extracellular adenosine triphosphate. Int. J. Oral Sci..

[62] Findley M.K., Koval M. (2009). Regulation and roles for claudin-family tight junction proteins. IUBMB Life.

[63] Barekatain R., Nattrass G., Tilbrook A.J., Chousalkar K., Gilani S. (2019). Reduced protein diet and amino acid concentration alter intestinal barrier function and performance of broiler chickens with or without synthetic glucocorticoid. Poult. Sci..

[64] Forder R.E., Nattrass G.S., Geier M.S., Hughes R.J., Hynd P.I. (2012). Quantitative analyses of genes associated with mucin synthesis of broiler chickens with induced necrotic enteritis. Poult. Sci..

[65] Cowieson A.J., Lu H., Ajuwon K.M., Knap I., Adeola O. (2017). Interactive effects of dietary protein source and exogenous protease on growth performance, immune competence and jejunal health of broiler chickens. Anim. Prod. Sci..

[66] Beam A., Clinger E., Hao L. (2021). Effect of diet and dietary components on the composition of the gut microbiota. Nutrients.

[67] Dong T.S., Luu K., Lagishetty V., Sedighian F., Woo S.L., Dreskin B.W., Katzka W., Chang C., Zhou Y., Arias-Jayo N. (2020). A high protein calorie restriction diet alters the gut microbiome in obesity. Nutrients.

[68] Masuoka H., Suda W., Tomitsuka E., Shindo C., Takayasu L., Horwood P., Greenhill A.R., Hattori M., Umezaki M., Hirayama K. (2020). The influences of low protein diet on the intestinal microbiota of mice. Sci. Rep..

[69] Cho S., Hwang O., Park S. (2015). Effect of dietary protein levels on composition of odorous compounds and bacterial ecology in pig manure. Asian-Australas. J. Anim. Sci..

[70] Lamendella R., Domingo J.W., Ghosh S., Martinson J., Oerther D.B. (2011). Comparative fecal metagenomics unveils unique functional capacity of the swine gut. BMC Microbiol..

[71] Ziemer C.J. (2013). Broad diversity and newly cultured bacterial isolates from enrichment of pig feces on complex polysaccharides. Microb. Ecol..

[72] Song M., Kim B., Cho J.H., Kyoung H., Choe J., Cho J.Y., Kim Y., Kim H.B., Lee J.J. (2022). Modification of gut microbiota and immune responses via dietary protease in soybean meal-based protein diets. J. Microbiol. Biotechnol..

[73] Dempsey E., Corr S.C. (2022). Lactobacillus spp. For gastrointestinal health: Current and future perspectives. Front. Immunol..

[74] Schaus S.R., Vasconcelos Pereira G., Luis A.S., Madlambayan E., Terrapon N., Ostrowski M.P., Jin C., Henrissat B., Hansson G.C., Martens E.C. (2024). Ruminococcus torques is a keystone degrader of intestinal mucin glycoprotein, releasing oligosaccharides used by bacteroides thetaiotaomicron. mBio.

[75] Liu H., Wang S., Chen M., Ji H., Zhang D. (2024). Effects of lactobacillus-fermented low-protein diets on the growth performance, nitrogen excretion, fecal microbiota and metabolomic profiles of finishing pigs. Sci. Rep..

[76] Zhang L., Ouyang Y., Li H., Shen L., Ni Y., Fang Q., Wu G., Qian L., Xiao Y., Zhang J. (2019). Metabolic phenotypes and the gut microbiota in response to dietary resistant starch type 2 in normal-weight subjects: A randomized crossover trial. Sci. Rep..

[77] Sharma M., Wasan A., Sharma R.K. (2021). Recent developments in probiotics: An emphasis on bifidobacterium. Food Biosci..

