# Supplementary material for: Protease and Bacillus coagulans Supplementation in a Low-Protein Diet Improves Broiler Growth, Promotes Amino Acid Transport Gene Activity, Strengthens Intestinal Barriers, and Alters the Cecal Microbial Composition
Source: Animals (Basel). 2025 Jan 10;15(2):170. doi: 10.3390/ani15020170 (PMC11758613; doi:10.3390/ani15020170)
Supplement: Supplementary file 1 [file animals-15-00170-s001.zip › animals-3347698-supplementary.pdf]

Table S1 The effect of protease and *Bacillus coagulans* supplementation to a low-protein diet on mortality rate in broilers

Items	CON	LPRO	PRO	PAB	SEM	*P*-value
mortality %	0.00	0.93	2.78	0.93	0.58	0.404
